# Central Venous Sinus Thrombosis in a Boy With Acute Severe Ulcerative Colitis

**DOI:** 10.3389/fped.2019.00019

**Published:** 2019-02-01

**Authors:** Rafael Martín-Masot, Pilar Ortiz Pérez, Juliana Serrano Nieto, María Martínez León, Antonia Pascual Martínez, Javier Blasco-Alonso, Victor Manuel Navas-López

**Affiliations:** ^1^Pediatric Gastroenterology and Nutrition Unit, Hospital Regional Universitario de Malaga, Málaga, Spain; ^2^Pediatric Radiology Department, Hospital Regional Universitario de Malaga, Málaga, Spain; ^3^Pediatric Hematology Department, Hospital Regional Universitario de Malaga, Málaga, Spain; ^4^Biomedical Research Institute of Málaga (IBIMA), University of Málaga, Málaga, Spain

**Keywords:** cerebral venous thrombosis (CVT), prophylaxis, inflammatory bowel disease, pediatrics, cerebral venous sinus thrombosis (CVST)

## Abstract

Cerebral venous sinus thrombosis (CVST) in childhood is uncommon. Certain diseases predispose patients to CVST, such as inflammatory bowel disease (IBD), which is considered a risk factor for developing thrombosis, which in turn is considered an extraintestinal manifestation of IBD. The use of prophylaxis in certain patients is a controversial topic. We present the case of a 5-years-old child with ulcerative colitis, who presented with transverse sinus thrombosis immediately after colectomy. Considering the recent recommendations on prophylaxis in this disease, our patient and probably many others would benefit from establishing treatment with low-molecular-weight heparin. We believe that these recommendations should be known, with our case serving as an example, given that we are heading in a direction that has so far been controversial.

## Background

Pediatric cerebral venous sinus thrombosis (CVST) has an incidence rate of 0.67 cases per 100,000 children and mainly occurs during the neonatal period (1). The diagnosis of CVST has increased due to greater diagnostic suspicion, improved neuroimaging techniques and increased survival of children with diseases that predispose them to the development of CVST. The most common risk factors in older children are head and neck infections and chronic systemic diseases such as chronic inflammatory and hematological diseases and cancer.

## Case Report

**Figure 1:**
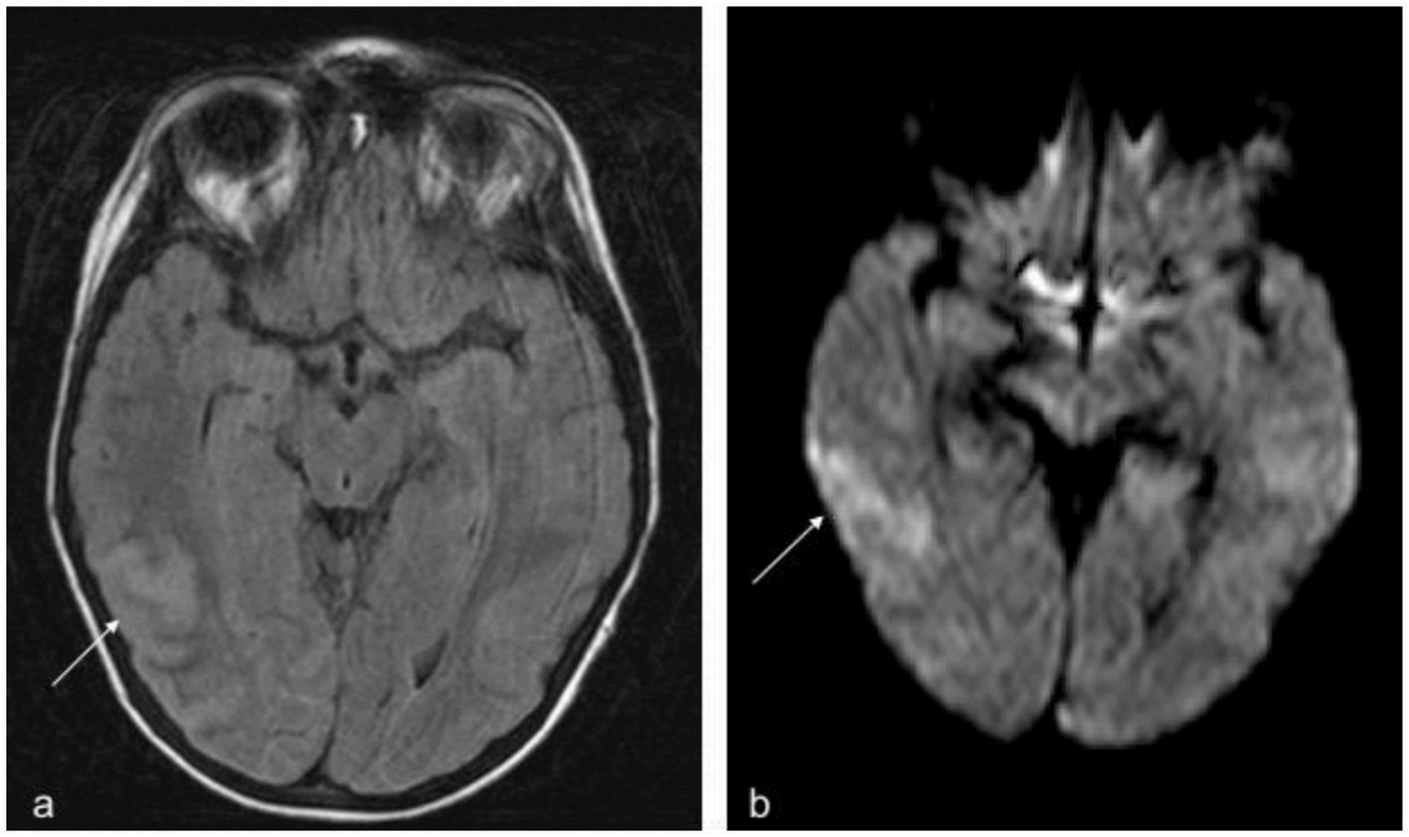
**(a)** Transverse plane, FLAIR sequence, movement artifacts. The arrow shows the cortical-subcortical lesion compatible with a non-haemorrhagic infarction in a venous territory. **(b)** Transverse plane, b1000 diffusion sequence. The arrow shows parenchymal shine due to diffusion restriction indicative of acute vascular disease (Not shown ADC map with signal drop in the area).

**Figure 2:**
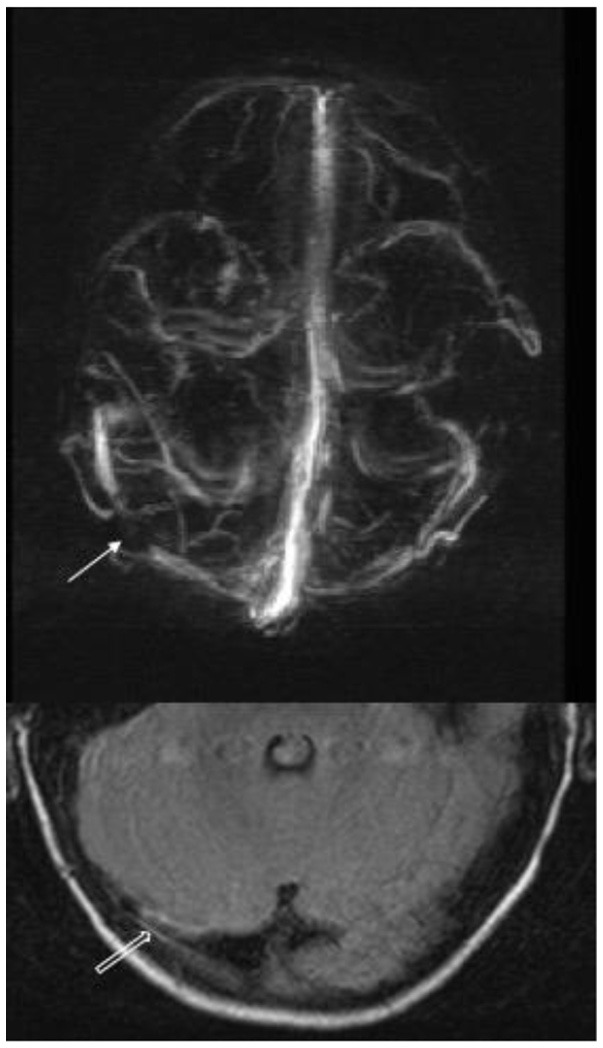
Venous Magnetic Resonance Angiography with contrast. 3D reconstruction in the transverse plane and magnification of the baseline sequence in the occipital region. The arrow shows the repletion defect caused by the thrombus in the middle third of the right transverse sinus, and the hollow arrow shows the peripheral wall contrast that delimits the luminal thrombosis of the sinus.

We present the case of a child aged 5 years and 2 months who was diagnosed at the age of 3 years with moderate (E4S0) ulcerative colitis (UC). After the initial presentation, the patient was started on treatment with oral mesalazine. In the following months, treatment with oral steroids was started, which was subsequently changed to intravenous, along with azathioprine, tacrolimus, and infliximab, without success. At 20 months from the disease onset, the treatment proceeded to the implementation of a total proctocolectomy with J-pouch reconstruction. The immediate postoperative period elapsed without incident and early total parenteral nutrition was started while he was fasting. However, 96 h after the surgery, the patient presented right hemicranial headaches, left arm distal monoparesis, a complex partial seizure, and subsequently two secondarily generalized seizures that ceased after the introduction of intravenous phenytoin. Based on the suspicion of a stroke, the patient underwent cranial Computed Tomography (CT), the results of which were normal. Magnetic Resonance Imaging (MRI) and Magnetic Resonance Angiography (MRA) for venous intracranial system (Figures 1, 2) revealed a right occipital venous infarction secondary to partial thrombosis of the right transverse sinus. The decision was made to start anticoagulant therapy with intravenous heparin and subsequently with low-molecular-weight heparin (LMWH). The patient presented no known cardiovascular risk factors, although a subsequent hypercoagulability study revealed that the patient was a homozygous carrier of the methylene tetrahydrofolate reductase (MTHFR) gene C677T mutation and that the parents were heterozygous carriers of this MTHFR mutation; the rest of the results of the study were normal. One month after the episode, the thrombosis image persisted in the MRA. The patient therefore underwent long-term therapy with acenocoumarol for 3 years. The patient's subsequent progress was satisfactory and no further treatment was necessary. The patient is now asymptomatic from the neurological and gastrointestinal point of view.

## Discussion

Strokes are 2 to 4 times more common in patients with inflammatory bowel disease (IBD) compared to the general population. It was documented that 1–7.7% of patients presented some event, while post-mortem studies reported that the figure can reach 40% of patients (2). Deep vein thrombosis and pulmonary embolism are the most commonly associated entities, the risk of which is especially high in young patients, women, smokers, those with S-protein deficiency, and patients with colonic disease (3). In particular, venous thrombotic phenomena are three times more common in patients with IBD than in healthy controls (3–5), with a prevalence of ~4% (6).

The relationship between venous thromboembolism and IBD is based mainly on the inflammatory component of IBD, which produces a hypercoagulability state with coagulation cascade activation, platelet activation, and fibrinolysis anomalies (4, 6, 7). IBD could be considered a risk factor *per se* for the development of venous thrombosis, which would be an extraintestinal manifestation of IBD (4).

The role of other risk factors in the development of thrombosis has been studied. The classic risk factors are also valid for this group, although, no differences in the prevalence of hereditary disorders predisposing to the development of thrombosis were shown compared to healthy controls. The study by Alatri et al. (6) found a statistically significant association between surgery and UC but not with Crohn's disease (CD), probably due to the greater inflammatory burden in patients with UC who undergo surgery compared with patients with CD who undergo surgery for fistulas or stenosis. No relationship with drugs was found, but a positive correlation with disease activity and hospitalization was reported (4).

The Canadian registry of ischemic infarction in the pediatric population identified a risk factor in 98% of children, a prothrombotic state in 41%, and a chronic systemic disease in 36% (1). In a meta-analysis of case-control studies published in 2010 including more than 200 cases of pediatric and neonatal venous sinus thrombosis and 1,200 controls, the prevalence of the factor V Leiden mutation and the prothrombin gene G20210A mutation was higher in cases than in the controls with a greater risk of developing CVST (8). In contrast, the value of the MTHFR gene 677CT mutation is controversial. A 2010 meta-analysis of case-control studies in adults reported comparable frequency of the MTHFR gene C677T mutation in 382 adult patients with CVST and in 1,217 controls (9) while, a 2011 meta-analysis reported a positive association between CVST and the MTHFR gene C677T mutation (10). Our patient's thrombophilia study documented that he is a homozygous carrier of the MTHFR gene C677T mutation, without presenting a factor V Leiden mutation or the prothrombin gene G20210A mutation. In our case, we can consider as risk factors the surgery in a patient with chronic IBD with a homozygous MTHFR gene C677T mutation, the insertion of a central venous access (for parenteral nutrition) and immobility.

From the clinical standpoint, venous thrombosis of the transverse sinus usually starts with headaches, which are frequently severe, and precede the other neurological signs. Focal abnormalities and seizures should alert physicians to the possibility of venous thrombosis. Although the clinical spectrum is variable, venous thrombosis should be suspected upon the onset of any neurological sign in patients with IBD, mainly in those with UC especially during the postoperative period (7).

CVST treatment should start as soon as possible after the diagnosis has been confirmed and should aim at the following: (a) reversing the predisposing factor when is known, (b) controlling the seizures and intracranial hypertension, (c) administering antithrombotic treatment to recanalize the sinus obstruction, (d) preventing the propagation of the thrombus, and (e) treating the prothrombotic state to prevent recurrences. Antithrombotic treatment during the acute phase of CVST in children is similar to that used in adults but has weaker evidence due to the lack of randomized studies in pediatric populations. The presence of a haemorrhagic venous infarction is not a contraindication for anticoagulant therapy. For children with CVST without significant intracranial hemorrhage, the evidence-based guidelines of the American College of Chest Physicians recommend initial treatment with unfractionated heparin or LMWH, followed by treatment with LMWH or vitamin K antagonists for at least 3 months (Grade 1B). Anticoagulation for 3 more months is recommended if the cerebral-sinus occlusion persists or if the symptoms progress (Grade 2C). For children with CVST and recurrent risk factors (e.g., nephrotic syndrome and undergoing treatment with asparaginase), antithrombotic prophylaxis is recommended due to the recurrence risk (Grade 2C). Thrombolysis, thrombectomy, and surgical decompression is reserved for cases of severe CVST that do not improve with initial treatment with unfractionated heparin (11). The American Heart Association and American Stroke Association guidelines published in 2011 on diagnosing and managing CVST, recommended treatment with full doses of LMWHs (even in the presence of cerebral hemorrhage) for pediatric patients older than 28 days followed by the treatment with LMWHs or oral vitamin K antagonists from 3 to 6 months (12).

The goal of long-term anticoagulant therapy is to prevent the recurrence of CVST (which can occur in 2–4% of patients) and of venous thrombosis in other locations (4–7%). A European cohort study of 396 patients with CVST, with a median age of 5 years and a 36-months follow-up, reported that venous thrombosis reoccurred in 22 children (6%) with 13 CVST (3%) at a mean of 6 months (range, 0.1–85). The recurrence of CVST was observed only in children with an initial CVST diagnosed after the age of 2 years. The independent factors associated with the recurrence of systemic or cerebral venous thrombosis in this pediatric population were the absence of anticoagulant therapy before the relapse, the persistence of the thrombosis in the neuroimaging control study and the presence of heterozygous prothrombin gene G20210A mutation (13). A mortality of 3% (12 patients) was observed in the first 2 weeks of the event.

There is controversy regarding the application of drug prophylaxis for the thrombosis. Various studies and guidelines have recommended prophylaxis with LMWH to prevent venous thromboembolism in adults with severe acute colitis (14, 15). This prophylaxis is not currently used routinely for pediatric patients. Although the absolute risk of venous thromboembolism is lower in children than in adults, it appears that the risk of the disease is greater compared to healthy pediatric controls (16). Lazzerini et al. (17) in a systematic review found that up to 50% of IBD patients who suffered an episode of thrombosis had at least one risk factor. A recent retrospective study, where data from 34,000 children who underwent abdominopelvic surgery were analyzed, showed an increasing rate of venous thrombotic episodes in IBD patients (0.98 vs. 0.19 %). Despite the identification of potential risk factors for the development of thrombosis, there are no evidence-based guidelines about the screening or prophylaxis of thrombosis in pediatric patients who are going to undergo abdominal surgery (18). Zitomerskyet et al. (19) and, more recently, Turner et al. recommended prophylaxis with heparin for this patient group; prophylaxis with LMWHs is recommended for patients with IBD if they present at least two of the following risk factors: tobacco use, oral contraceptives, complete immobility, central venous catheters, obesity, concomitant infection, previous venous thromboembolic event, family history, and known prothrombotic disease (20). A systematic review of the literature (21) on the need for preoperative thromboprophylaxis in patients with IBD, recommends with grade III quality of evidence (according to the GRADE system, i.e., evidence from non-experimental descriptive studies, such as comparative studies and case–control studies), the need for pre and postoperative thromboprophylaxis in patients with IBD who are not at risk of bleeding, in order to prevent the risk of thrombosis. Previously, the Canadian Gastroenterology Association (22) had recommended against routine thromboprophylaxis in pediatric patients hospitalized for a flare of IBD, but recommended prophylaxis only in obese adolescents with severe acute colitis undergoing surgery and in hospitalized patients with a previous history of thrombosis.

These recommendations are similar to those proposed by Turner et al. (23) since they combine more than one risk factor for the development of thrombosis. Patients with IBD and severe acute colitis, would probably benefit from screening prior to the surgery, considering that acute colitis *per se* is a risk factor for the development of thrombosis and that the presence of a genetic prothrombotic factor is unknown, although cost-benefit studies are necessary. If prophylaxis is established, enoxaparin 1 mg/kg/day (100 IU/kg/day) in one daily dose is the most recommended drug (23).

In our case, the patient presented two risk factors for developing thrombosis: central venous catheter (for total parenteral nutrition) and prolonged immobility. Considering the new criteria proposed by the authors, prophylactic treatment should have therefore been started but this is not what happened with our patient who revealed at his subsequent examination, the presence of a homozygous MTHFR gene C677T mutation.

Several studies have been conducted in the past in order to evaluate the risk of bleeding and other complications derived from thromboprophylaxis. Some studies, carried out in pediatric population, concluded that thromboprophylaxis is safe and well tolerated with a bleeding rate of 1–5% (24, 25). In previous studies (1, 26, 27), patients with venous sinus thrombosis who received heparin did not have more bleeding episodes or worsening, compared with the placebo group, while mortality was also lower in the treatment group (28–30). Finally, a systematic review and meta-analysis of the literature, with more than 2,000 pediatric patients included, suggested that thromboprophylaxis in pediatric age is safe and effective (31).

Although CVST is a rare (even more so in childhood) but potentially severe complication, we believe it is important to understand the association, especially in patients who undergo surgery, because the neurological prognosis will depend on early diagnosis and early initiation of treatment.

Although the use of prophylaxis is not currently standardized, the implementation of the new guidelines and further studies are expected to change the clinical practice. Although thromboprophylaxis is safe and effective, the risk of thrombosis and bleeding requires individual assessment to optimize the treatment and minimize the complications, costs, and outcome of the disease.

## Ethics Statement

Written informed consent was obtained from parents before manuscript preparation and submission.

## Author Contributions

All authors listed have made a substantial, direct and intellectual contribution to the work, and approved it for publication.

### Conflict of Interest Statement

The authors declare that the research was conducted in the absence of any commercial or financial relationships that could be construed as a potential conflict of interest.
